# 2,3,5,4′-Tetrahydroxystilbene-2-O-β-D-glucoside (TSG) from *Polygonum multiflorum* Thunb.: A Systematic Review on Anti-Aging

**DOI:** 10.3390/ijms26073381

**Published:** 2025-04-04

**Authors:** Can Zhu, Jinhong Li, Wenchao Tang, Yaofeng Li, Chang Lin, Danhong Peng, Changfu Yang

**Affiliations:** 1 College of Basic Medicine, Guizhou University of Traditional Chinese Medicine, Guiyang 550025, China; 2 College of Pharmacy, Guizhou University of Traditional Chinese Medicine, Guiyang 550025, China

**Keywords:** 2,3,5,4′-tetrahydroxystilbene-2-O-β-D-glucoside (TSG), aging, age-related diseases, *Polygonum multiflorum*, healthy longevity

## Abstract

The global rise in aging populations has made healthy longevity a critical priority in medical research. 2,3,5,4′-Tetrahydroxystilbene-2-O-β-D-glucoside (TSG), the primary bioactive component of *Polygonum multiflorum* Thunb. (commonly known as *Fallopia multiflora* Thunb., He shou wu, Fo-ti, or Polygoni multiflori radix), has emerged as a promising agent for combating aging and age-related diseases. This systematic review evaluates the anti-aging properties of TSG and its protective effects against age-related pathologies. The current evidence demonstrates that TSG exhibits comprehensive anti-aging effects, including lifespan extension, neuroprotection (e.g., ameliorating Alzheimer’s and Parkinson’s diseases), cardiovascular protection (e.g., reducing atherosclerosis and hypertension), delay of gonadal aging, reduction in bone loss (e.g., mitigating osteoporosis), and promotion of hair regrowth. Mechanistically, TSG alleviates oxidative stress, inflammation, and apoptosis while enhancing mitophagy, mitochondrial function telomerase activity, and epigenetic regulation. These multi-target actions align with the holistic principles of traditional Chinese medicine, highlighting TSG’s potential as a multifaceted anti-aging agent. However, further research is required to establish standardized quantitative systems for evaluating TSG’s efficacy, paving the way for its broader clinical application in promoting healthy aging.

## 1. Introduction

Aging is characterized by the progressive decline of organismic functions in adulthood, leading to increased vulnerability to neurodegenerative diseases, cardiovascular disorders, cancer, and ultimately death [1,2]. In recent years, significant progress has been made in aging research. In 2013, nine hallmarks of aging were proposed, including genomic instability, telomere attrition, epigenetic alterations, loss of proteostasis, deregulated nutrient sensing, mitochondrial dysfunction, cellular senescence, stem cell exhaustion, and altered intercellular communication [3]. These hallmarks were later expanded to twelve in 2023, with the addition of disabled macroautophagy, chronic inflammation, and dysbiosis [4]. These characteristics are interconnected and contribute to the development of age-related diseases. For instance, DNA damage triggers inflammation through signaling cascades, driving multiple age-related pathologies [5]. Similarly, oxidative stress interacts with the epigenome, further exacerbating age-related conditions [6]. Additionally, factors secreted by senescent cells promote chronic inflammation, which in turn accelerates cellular senescence, creating a vicious cycle that fuels aging and its associated diseases [7]. With aging populations growing worldwide, developing interventions to promote healthy longevity has become a paramount biomedical priority.

**Figure 1:**
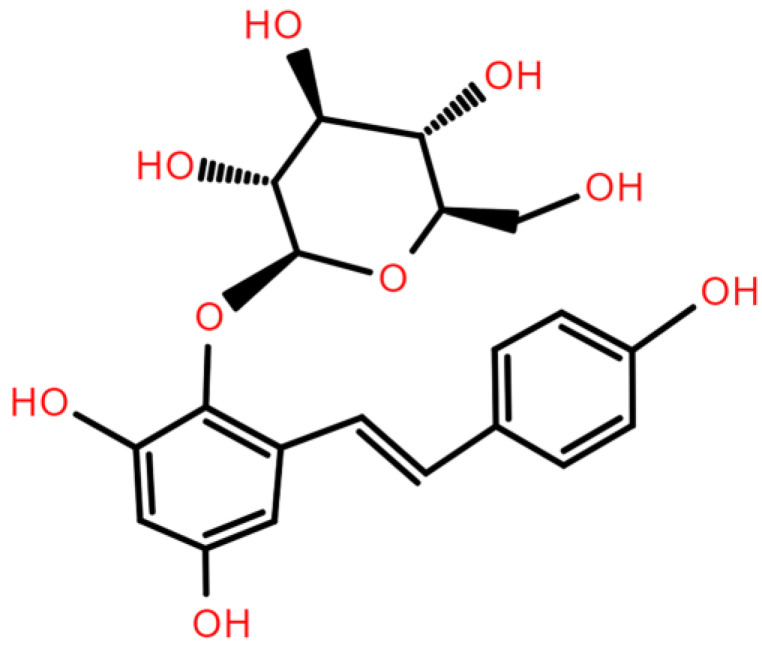
Chemical structure of TSG.

2,3,5,4′-Tetrahydroxystilbene-2-O-β-D-glucoside (TSG, Figure 1), the predominant bioactive component of *Polygonum multiflorum* Thunb. (*P. multiflorum;* commonly known as *Fallopia multiflora* Thunb., He shou wu, Fo-ti, or Polygoni multiflori radix), can be obtained through various extraction and purification approaches. Conventional extraction methods include aqueous decoction and ethanol reflux. Subsequent purification is commonly achieved through chromatographic separation or recrystallization. First documented in the Pharmacopoeia of China (1963) as the primary quality control marker [8], TSG has garnered attention for its potential anti-aging properties. Modern pharmacological studies have demonstrated that TSG exhibits considerable potential in mitigating aging and age-related diseases. However, the mechanisms underlying these effects remain fragmented, and comprehensive reviews focusing on TSG are limited. This review systematically consolidates the latest findings on TSG’s anti-aging properties and its protective effects against age-related pathologies, elucidating its pleiotropic mechanisms and therapeutic potential to inform future research on healthy aging interventions. Additionally, we also briefly evaluate the anti-aging effects of bioactive constituents in *P. multiflorum* beyond TSG, providing insights into the potential synergistic action of the said compound with other plant constituents.

## 2. Methods

### 2.1. Search Strategy

A comprehensive literature search was conducted across five databases, including PubMed, Embase, the Chinese National Knowledge Infrastructure (CNKI), the Chinese Biomedical Database (CBM), and the Chinese Technology Periodical Database, from their inception to 31 December 2024. The search focused on original articles published within the last five years to ensure the inclusion of the most recent findings. The search strategy utilized a combination of MeSH terms and free-text keywords, as detailed in Supplementary Table S1.

### 2.2. Eligibility and Study Selection

**Figure 2:**
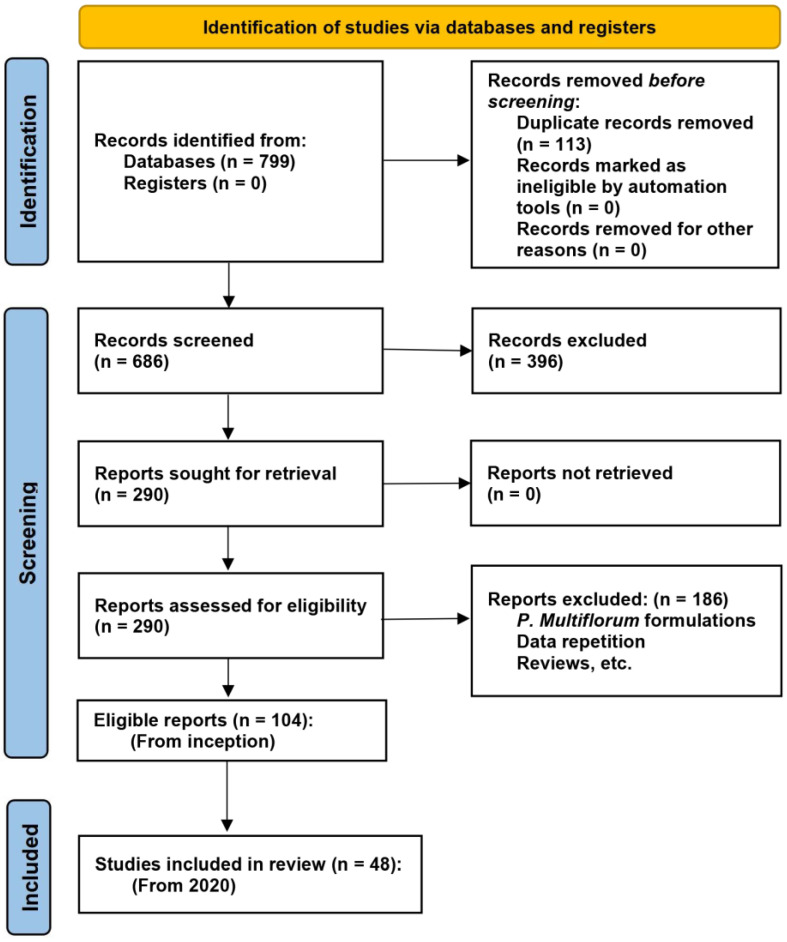
PRISMA 2020 flow diagram for new systematic reviews.

Figure 2 illustrates the study selection process. Following the screening of titles and abstracts, 290 studies were identified for full-text retrieval. After a thorough eligibility assessment, 186 studies were excluded because, e.g., they involved *P. multiflorum* formulations or were review articles. Consequently, 104 reports were deemed eligible for inclusion in this systematic review, encompassing studies from the inception of the databases to the present. Among these, 48 studies published within the last five years (2020–2024) were included in the final analysis. These studies were categorized by their primary focus: lifespan extension (n = 4), neuroprotection (n = 19), cardiovascular protection (n = 10), reproductive protection (n = 5), bone protection (n = 4), and others (n = 6). This selection ensured a comprehensive and up-to-date evaluation of the potential anti-aging benefits of TSG and other extracts from *P. multiflorum*.

The study selection process was conducted independently by two or three authors, with any disagreements resolved through discussion and consensus involving the corresponding authors. For studies included in the final analysis, a standardized data extraction form was developed to systematically collect key information from each study.

## 3. Results and Discussion

**Figure 3:**
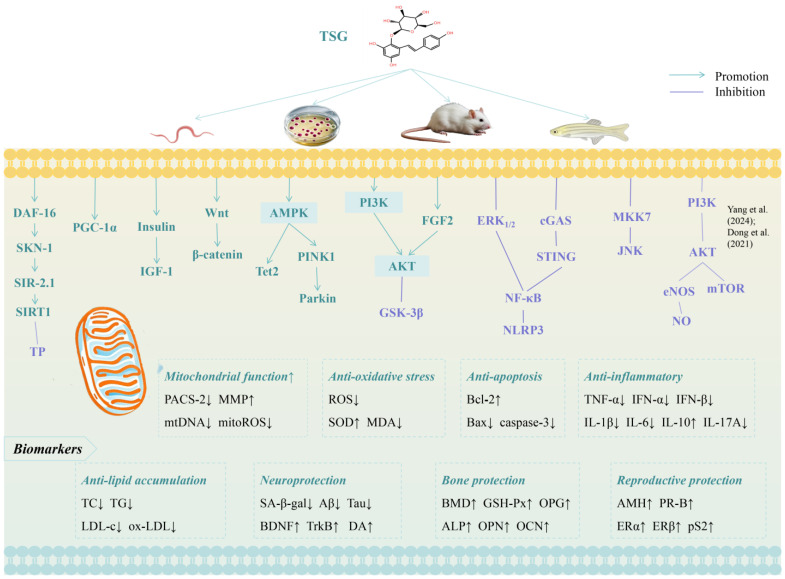
Molecular mechanisms of TSG against aging [25,32]. Arrow symbols denote: (↑) upregulation, (↓) downregulation.

**Figure 4:**
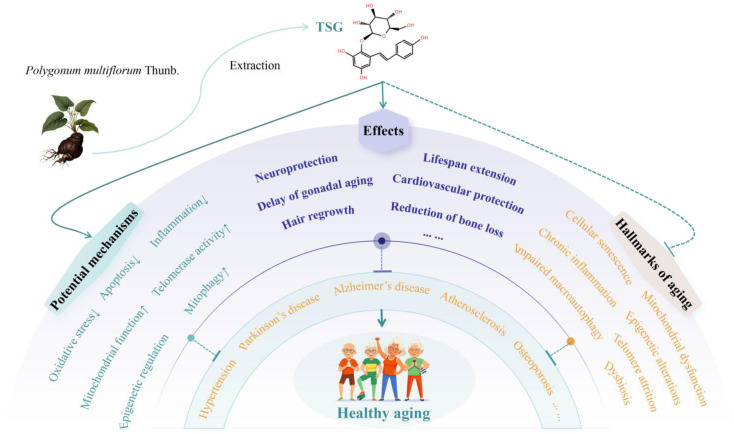
Mechanisms and therapeutic effects of TSG in promoting healthy longevity. Arrow symbols denote: (↑) upregulation, (↓) downregulation.

Table 1 summarizes the recent studies investigating the effects of TSG against aging and age-related diseases. Figure 3 and Figure 4 illustrate the molecular mechanism and therapeutic effects of TSG in promoting healthy longevity.

**Table 1:** Summary of recent studies (2020–2024) on the effects of TSG against aging and age-related diseases.

Effects	Aging Model (Inducer; Object)	TSG Treatment (Dose; Duration)	Chemical Purity	Potential Mechanisms	Author (Year)	References
Lifespan extension	H_2_O_2_; larval zebrafish	25, 50, and 100 μg/mL; 24 h	>98%	Oxidative stress↓, inflammation↓ (SA-β-gal↓, ROS↓, SOD↑, catalase↑, *il-1β*↓, *il-6*↓, *cxcl-c1c*↓, *il-8*↓)	Xia et al. (2023)	[9]
	*C. elegans*	100, 200, and 400 μM; until death	Unclear (standard)	Mean lifespan↑, mitochondrial function↑ (DAF-16/SKN-1/SIR-2.1 pathways; DAF-16↑, SKN-1↑, SIR-2.1↑, SIRT1↑, Aβ↓, Tau↓, ROS↓, MMP↑)	Sun et al. (2024)	[10]
Neuroprotection	Radiation; C57BL/6J mice, or *Tet2^−/−^* mice	40, 80, and 120 mg/kg/d; 5 months	98%	Inflammation↓, neurogenesis↑ (AMPK/Tet2 pathway; AMPK↑, Tet2↑, NLRP3↓)	Miao et al. (2022)	[11]
	LPS/ATP + Aβ; BV2, N2a, and SH-SY5Y cells, and primary microglia	10, 100 nM, and 1, 10 μM; 24 h	>98%	Inflammation↓, mitophagy↑, mitosis↑ (AMPK/PINK1/Parkin pathway; AMPK↑, PINK1↑, Parkin↑, NLRP3↓, LC3-II/LC3-I↑, p62↓; iNOS↓, COX-2↓, Drp1↑, MIRO↓, Mfn2↑, MFF↓)	Gao et al. (2020)	[12]
	Okadaic acid; SH⁃SY5Y cells	100 μM; 24 h	Unclear (standard)	Apoptosis↓ (PI3K/AKT pathway; PI3K↑, AKT↑, Bcl⁃2↑, Bax↓)	Kang et al. (2024)	[13]
	High-glucose; HT-22 cells	200 µM; 48 h	Unclear (standard)	Apoptosis↓ (HAT↓, HDAC↑, Bcl-2↑, Bax↓, caspase-3↓)	Chen et al. (2022)	[14]
Alleviating AD	① AD model: APP/PS1 double transgenic mice ② LPS/IFN-γ; BV2 cells	① 40 and 80 mg/kg/d; 5 months ② 25, 50, and 100 μM; 20 h	Unclear (standard)	Inflammation↓ (cGAS-STING pathway; cGAS↓, STING↓, NF-κB↓, NLRP3↓; IL-1β↓, IL-6↓, TNF-α↓, IFN-α↓, IFN-β↓, IFIT1↓, IRF7↓)	Gao et al. (2023)	[15]
	AD model: APP/PS1/Tau triple transgenic mice	0.033, 0.1, and 0.3 g/kg/d; 60 days	≥70%	CDK5↓, MAPK1↓, PP1↑, Tau↓, p39↓	Wu et al. (2022)	[16]
	AD model: Aβ_25–35_; SD rats	0.033, 0.1, and 0.3 g/kg; 4 or 8 weeks	98%	Apoptosis↓, improving neuronal morphology (PI3K/AKT/GSK-3β pathway; MKK7/JNK pathway; PI3K↑, AKT↑, GSK-3β↓, Tau↓; MKK7↓, JNK↓)	Xia et al. (2023); Li, YB et al. (2023); Li, Y et al. (2023)	[17,18,19]
	AD model: N2a/APP695swe cells	100 μM; 48h	98%	Apoptosis↓, improving mitochondrial function (MMP↑; PACS-2↓)	Wang et al. (2024)	[20]
	AD model: APP/PS1 mouse	120 mg/kg; 8 weeks	Unclear (standard)	MAPK pathway, chemokine pathway and autophagy—animal	Gao et al. (2024)	[21]
Ameliorating PD	① PD model: MPTP; C57BL/6J mice ② MPP+; mesencephalic DA neurons or SH-SY5Y cells	① 20 mg/kg; 7 days ② unclear	≥98%	Apoptosis↓, neurotoxicity↓ (FGF2-Akt, BDNF-TrkB axis; FGF2↑, Akt↑, DA↑, TH↑, BDNF↑, TrkB↑, Bcl⁃2↑, caspase-3↓)	Yu et al. (2019)	[22]
	Mouse neural stem cells	10 μM; 2 weeks	≥98%	DA neuron differentiation (Wnt/β-catenin pathway; Wnt1↑, Wnt3a↑, Wnt5a↑, β-catenin↑, Nurr1↑)	Zhang et al. (2021)	[23]
Inhibiting AS	High-fat diet; ApoE-deficient (ApoE^−/−^) mice	0.035 and 0.07 mg/g/d; 8 weeks	≥98%	Inflammation↓, lipid accumulation↓, AS plaque↓, and regulating intestinal microbiota (TG↓, ox-LDL↓, IL- 6↓, TNF-α↓, VCAM-1↓, MCP-1↓)	Li et al. (2020)	[24]
	① ox-LDL; BMDCs ② ApoE^−/−^ mice	① 40 and 80 µM, 2 h ② 40 mg/kg/d, 5 weeks	≥98%	Autophagy↓, DCs maturation↓, Treg differentiation↑, inflammation↓, lipid accumulation↓, (PI3K/AKT/mTOR pathway; PI3K↓, AKT↓, mTOR↓, P62↓; TC↓, TG↓, LDL-C↓; IL-6↓, IL-17A↓, IL-10↑)	Yang et al. (2024)	[25]
	① ApoE^−/−^ mice ② Macrophages in the aorta cells of mice (in ①)	100 mg/kg/d, 8 weeks	99%	Atherosclerotic lesions↓, dyslipidemia symptoms↓, and regulating lipid metabolism (*Srepb-1c*↓, *Fasn*↓, *Scd1*↓, *Gpat1*↓, *Dgat1*↓, *Pparα*↑ and *Cpt1α*↑; *Srebp2*↓, *Hmgcr*↓, *Ldlr*↑, *Acat1*↓, *Acat2*↓, and *Cyp7a1*↑)	Li et al. (2024)	[26]
	① High-fat diet; LDLr^−/−^ mice ② ox-LDL; HAECs	① 50 and 100 mg/kg/d; 12 weeks ② 1, 10, and 100 μM; 24 h	>98%	Oxidative stress↓, endothelial senescence↓, telomerase activity↑, mitochondrial damage↓, and improving lipid profiles (PGC-1α pathway; PGC-1α↑, TC↓, TG↓, LDL-c↓, ox-LDL↓; γ-H2AX↓, p53↓, p21↓, p16↓; TERT↑; mitoROS↓, NRF1↑, TFAM↑; ROS↓, MDA↓; β-gal↓, MMP↑, mtDNA↓, SOD↑, CAT↑)	Wang et al. (2022)	[27]
Cardiovascular protection	① Natural aging C57BL/6J mice, *Tet2* Mut mice ② IMR-90 fibroblasts	① 120 mg/kg/d; 60 days ② 10 and 100 μM; 48 h	Unclear (standard)	HSC aging↓, repopulation potential↑, epigenetic reprograming↑, stemness↑ (AMPK-Tet2 axis; CLPs↑, B lymphocytes↑)	Gao et al. (2024)	[28]
	Ang Ⅱ; HUVECs	50 and 100 μM; 24 h	Unclear (standard)	Endothelial senescence↓ (SA-β-gal↓, p53↓, PAI-1↓, SIRT1↑)	Fan et al. (2021)	[29]
Anti-hypertension	U46619; superior mesenteric artery of SD rats	Concentration accumulation: 10^−5^ ∼10^−2^ M;	≥98%	Vasodilation (SIRT1/TP pathway; SIRT1↑, TP↓)	Chen et al. (2022)	[30]
	HHcy; C57BL/6 mice	40, 80, and 160 mg/kg; 4 weeks	98%	Inhibiting vasoconstriction (ERK_1/2_/NF-κB pathway; p-ERK_1/2_↓, p-p65↓, endothelin-1↓; BP↓, Hcy↓)	Jia et al. (2022)	[31]
	① ZDF rats, OMT^−/−^ mice ② HUVEC and mature adipocyte co-culture	① 50, 100, and 200 mg/kg/d; 2 weeks ② 100 μM; 24 h	≥98%	Oxidative/nitrative stress↓, improving endothelial function (Akt/eNOS/NO pathway; SBP↓, omentin-1↑, Akt↓, eNOS↓, NO↓; NOX2↓, p22^phox^↓, SOD↓, peroxynitrite anion↓, PPAR-γ↑, *Itln-1*↑)	Dong et al. (2021)	[32]
Reproductive protection	H_2_O_2_ + FeSO_4_; rat testicular Leydig cells	150 μM; 48 h	Unclear (standard)	Oxidative stress↓, cell senescence↓ (Insulin/IGF-1pathway; SA-β-gal↓, IRS1↑, IGF-1↑, IRS2↑, INSR↑, IGFBP3↓)	Li et al. (2021)	[33]
	Normal C57BL/6J mice	10 mg/kg/d; 32, or 16 weeks	95%	Oocyte quantity and quality↑, mitochondrial biogenesis↑, steroidogenesis ↑ (AMH↑, PR-B↑, *atp6*↑, *pgc1α*↑, *cyp11a*↑, *cyp19*↑, *er-β*↑)	Lin et al. (2022)	[34]
Estrogenic activity	ER (+) MCF-7 cells	100 nM; 24 h	Unclear (standard)	Cell proliferation↑, acting as phytoestrogen (ERα↑, ERβ↑, pS2↑)	Akter et al. (2023)	[35]
Reducing OP	① OP model: OVX; SD rats ② H_2_O_2_; MC3T3-E1 cells	① 80 mg/kg/d; 3 months ② 10 μM; 24 h	≥98%	Oxidative stress↑, apoptosis↓, bone resorption↓ (miR-34a↑, SIRT1↓; Conn.D↑, Tb.N↑, BMD↑, MDA↓, GSH-Px↑; ALP↑, OPN↑, COL-1↑, OCN↑)	Wang et al. (2022)	[36]
	Diabetic OP model: Streptozotocin; C57BL/6J mice	10 and 40 mg/kg; 8 weeks	Unclear (standard)	Regulating osteogenesis and osteoclast genesis (Ca↑, RUNX-2↑, COL-I↑, OCN↑, β-catenin↑, RAS↓, OPG↑, RANKL↓, sclerostin↓)	Zhang et al. (2019)	[37]
Bone protection	BMSCs	10^−6^, 10^−5^, and 10^−4^ M; 3 or 7 days	Unclear (standard)	Cell proliferation↑, osteogenic differentiation↑ (ALP↑, OCN↑, Col1a1↑, Runx2↑, β-catenin↑)	Liang et al. (2022)	[38]

Note: Arrow symbols denote: (↑) upregulation, (↓) downregulation.

### 3.1. Lifespan-Extending Effects of TSG

Under laboratory conditions, the lifespan of *Caenorhabditis elegans* (*C. elegans*) is approximately three weeks, making it a widely used model organism for lifespan studies. TSG has demonstrated significant antioxidative and lifespan-extending properties. For instance, in *C. elegans*, TSG was shown to enhance the resistance to lethal thermal stress and effectively prolong both the mean and maximum lifespan of these organisms [39]. Similarly, in *Drosophila melanogaster*, TSG extended lifespan and improved climbing ability, further supporting its anti-aging potential [40].

In a study involving H_2_O_2_-induced aging in larval zebrafish, TSG pretreatment reduced the activity of senescence-associated β-galactosidase (SA-β-gal), inhibited the accumulation of reactive oxygen species (ROS), and enhanced the activity of antioxidant enzymes such as superoxide dismutase (SOD) and catalase [9]. Furthermore, TSG suppressed the expression of inflammation-related genes *(il-1β*, *il-6*, and *il-8*), collectively protecting against oxidative-stress-mediated aging in zebrafish [9].

Recent research has further elucidated the mechanisms underlying TSG’s lifespan-extending effects. In *C. elegans*, treatment with 200 μM TSG increased the mean lifespan by 16.48% and delayed age-associated physiological decline [10]. This effect was mediated through the regulation of mitochondrial quality control processes, involving key pathways such as abnormal dauer formation-16 (DAF-16)/forkhead box O (FOXO), skinhead-1 (SKN-1)/nuclear factor erythroid 2-related factor 2 (Nrf2), and silent information regulator-2.1 (SIR-2.1)/sirtuin 1 (SIRT1), which collectively improve mitochondrial function [10].

As the principal bioactive compound and quality marker of *P. multiflorum*, TSG’s lifespan-extending effects align with the traditional pharmacological view that *P. multiflorum* can “prolong lifespan”. These effects are likely attributed to its antioxidative, anti-inflammatory, and mitochondrial function-enhancing properties, thereby providing a scientific basis for the anti-aging effects of *P. multiflorum*.

### 3.2. Neuroprotective Effects of TSG

TSG has demonstrated significant neuroprotective effects through multiple mechanisms. In vitro experiments revealed that TSG enhanced mitophagy and mitochondrial function via the AMP-activated protein kinase (AMPK)/PTEN-induced kinase 1 (PINK1)/Parkin pathway, effectively mitigating neuroinflammation induced by lipopolysaccharide (LPS)/ATP and β-amyloid (Aβ) [12]. Additionally, TSG modulated apoptotic pathways by upregulating histone deacetylase (HDAC) and B-cell lymphoma 2 (Bcl-2) while downregulating histone acetyltransferase (HAT), Bcl-2-related X protein (Bax), and caspase-3. These changes improved the survival of mouse hippocampal neuron HT-22 cells under high-glucose conditions [14]. Furthermore, TSG activated the phosphatidylinositol-3-kinase (PI3K)/protein kinase B (AKT) signaling pathway, leading to increased Bcl-2 levels and reduced Bax expression, thereby attenuating okadaic acid-induced apoptosis in human neuroblastoma SH-SY5Y cells [13]. TSG also delayed cellular senescence in SH-SY5Y cells, potentially through the regulation of apoptosis-related proteins by *Gstm3* [41]. Moreover, TSG promoted neurotrophic support by significantly increasing the expression of brain-derived neurotrophic factor (BDNF), glial-derived neurotrophic factor (GDNF), and nerve growth factor (NGF) in rat primary astroglia, accompanied by enhanced phosphorylation of extracellular signal-regulated kinase (ERK) 1/2 [42]. This suggests that TSG stimulates the release of astroglia-derived neurotrophic factors, highlighting its therapeutic potential for neurological disorders.

In vivo, TSG activated AMPK and modulated the AMPK/ten–eleven translocation methylcytosine dioxygenase 2 (Tet2) pathways, promoting neural precursor cell proliferation and differentiation while suppressing NOD-like receptor protein 3 (NLRP3) inflammasome activation in microglia. These mechanisms collectively contributed to the prevention of radiation-induced cognitive deficits in mice [11].

In summary, TSG exhibits robust neuroprotective properties, enhancing neuronal survival and cognitive function. However, the specific types of neurodegenerative diseases addressed in these studies remain unclear. The following sections will explore the preventive and therapeutic potential of TSG in common neurodegenerative disorders.

#### 3.2.1. Attenuation of Alzheimer’s Disease (AD)

Alzheimer’s disease (AD), one of the most prevalent neurodegenerative disorders, exhibits an age-dependent increase in incidence [43]. TSG demonstrated significant efficacy in enhancing both spatial and non-spatial learning and memory capabilities in APPswe/PS1dE9 (APP/PS1) double transgenic AD model mice. This neuroprotective effect was mediated by the inhibition of microglia activation and inflammatory cytokine expression through the cyclic GMP-AMP synthase (cGAS)/stimulator of interferon genes (STING) signaling pathway, both in vitro and in vivo [15]. Furthermore, TSG exerted beneficial effects in APP/PS1/Tau triple transgenic AD mice, primarily through the downregulation of cyclin-dependent kinase 5 (CDK5) and mitogen-activated protein kinase 1 (MAPK1), coupled with the upregulation of protein phosphatase 1 (PP1) and the inhibition of Tau protein phosphorylation [16]. TSG also demonstrated protective effects by downregulating phosphofurin acidic cluster sorting protein-2 (PACS-2), which reduced apoptosis in N2a/APP695swe model cells and enhanced the mitochondrial membrane potential (MMP) [20]. In an Aβ_25-35_-induced AD rat model, TSG inhibited neuron apoptosis in the hippocampal and cortical regions, improving neuronal morphology. This effect was potentially mediated by the activation of the PI3K/AKT signaling pathway, which inhibited glycogen synthase kinase-3β (GSK-3β), ultimately reducing Tau protein phosphorylation [17,18].

Additionally, TSG’s neuroprotective effects on neuronal injury repair were linked to the downregulation of the MKK7/jun N-terminal kinase (JNK) pathway [19]. TSG also mitigated AD progression by enhancing mitochondrial function, reducing Aβ production, and increasing neurotrophic factor levels [44]. Network pharmacology and molecular docking analyses suggested that TSG’s therapeutic mechanisms in AD primarily involved neuroprotection, anti-inflammatory effects, and the modulation of aging-related processes [45]. Recent research employing data-independent acquisition (DIA)-based quantitative proteomics has identified candidate protein biomarkers in the brain tissues of APP/PS1 transgenic mice following TSG treatment; pathway enrichment analysis identified several biologically relevant pathways associated with these changes, including neurodegenerative disease pathways (AD, PD, and Huntington’s disease), and cellular signaling pathways (MAPK signaling, chemokine signaling, and autophagy regulation) [21].

Accordingly, TSG shows significant potential in alleviating AD by inhibiting inflammation and apoptosis, reducing Aβ production, enhancing mitochondrial function and elevating neurotrophic factor levels, thus improving learning-memory deficits and cognitive impairment.

#### 3.2.2. Amelioration of Parkinson’s Disease (PD)

Parkinson’s disease (PD) is a common neurodegenerative disorder characterized by the progressive loss of dopaminergic (DA) neurons in the mesencephalic substantia nigra [46]. Studies indicated that TSG could restore the fibroblast growth factor 2 (FGF2)/Akt and BDNF/tropomyosin receptor kinase-B (TrkB) signaling axes, as well as protect DA neurons. This neuroprotective effect significantly mitigated neurotoxicity in both the 1-methyl-4-phenyl-1, 2, 3, 6-tetrahydropyridine (MPTP)-induced PD mouse model and the 1-methyl-4-phenylpyridinium (MPP+)-mediated SH-SY5Y cell line [22]. In experiments involving A53T mutant α-synuclein-transfected cells (A53T AS cells) exposed to MPP+, TSG pretreatment markedly enhanced cell viability and MMP. It also inhibited the overexpression and aggregation of α-synuclein while reducing ROS levels, the Bax/Bcl-2 ratio, and caspase-3 activity [47]. Additionally, TSG exhibited protective effects against DA neurons in 6-hydroxydopamine (6-OHDA)-induced neurotoxicity models by suppressing microglial activation and the subsequent release of pro-inflammatory factors [48]. Moreover, TSG might promote the differentiation of mouse neural stem cells (NSCs) into DA neurons by activating the Wnt/β-catenin signaling pathway, highlighting its potential therapeutic role in PD through NSC transplantation [23].

Therefore, TSG exerts neuroprotective effects by attenuating the neurotoxicity of MPP+, MPTP, or 6-OHDA, suppressing oxidative stress and inflammation, reducing cell apoptosis, and potentially facilitating the differentiation of NSCs into DA neurons. These mechanisms underscore TSG’s potential as a promising therapeutic agent for mitigating PD progression.

### 3.3. Cardiovascular Protective Effects of TSG

#### 3.3.1. Inhibition of Vascular Senescence and Atherosclerosis (AS)

Epidemiological studies consistently identify age as a major cardiovascular risk factor, with vascular aging closely linked to age-related macrovascular diseases such as atherosclerosis (AS) [49]. AS is characterized by the pathological deposition of lipids, thromboses, connective tissues, and calcium in the vascular system. TSG has been shown to significantly improve endothelial function through enhancement of the NO-cGMP pathway in ApoE-deficient (ApoE^−/−^) mice, suggesting its potential vasoprotective effects and therapeutic role in endothelial dysfunction-related vascular diseases, including AS [50].

In studies using oxidized low-density lipoprotein (ox-LDL) to stimulate human aortic endothelial cells (HAECs), TSG could alleviate endothelial aging, telomere dysfunction, oxidative stress, and mitochondrial damage, partly through the activation of the peroxisome proliferator-activated receptor-gamma coactivator-1alpha (PGC-1α) pathway, as supported by in vivo and in vitro evidence [27]. Furthermore, TSG markedly inhibited AS plaque formation in ApoE^−/−^ mice by improving lipid metabolism, reducing TG and ox-LDL levels, and suppressing inflammation through the downregulation of serum IL-6, tumor necrosis factor-α (TNF-α), vascular cell adhesion molecule-1 (VCAM-1), and monocyte chemotactic protein-1 (MCP-1) [24]. It also modulated intestinal microbiota, influencing *Firmicutes*, *Bacteroidetes*, and *Helicobacter pylori*, which collectively contributed to its anti-AS effects [24].

TSG regulated immune responses by reducing dendritic cell (DC) maturation and promoting Treg differentiation, correcting the Treg/Th17 imbalance via inhibition of the PI3K-AKT-mTOR signaling pathway. This immune modulation was mediated by TSG-induced DC lipophagy, which reduced lipid accumulation and inflammation [25]. In ApoE^−/−^ mice fed a high-fat diet, TSG lowered serum lipids, restored Treg/Th17 balance, and reduced pro-inflammatory cytokines while increasing anti-inflammatory factors, correlating with decreased arterial DC P62 content and plaque area, further supporting TSG’s anti-AS potential [25]. At the metabolic level, TSG restored hepatic lipid metabolism by regulating fatty acid and cholesterol metabolism-related genes, while promoting the polarization of aortic macrophages to the M2 phenotype, further alleviating AS progression [26].

In studies involving angiotensin II (Ang II)-incubated human umbilical vein endothelial cells (HUVECs), TSG pretreatment reduced senescence markers such as SA-β-gal, p53, and plasminogen activator inhibitor-1 (PAI-1), indicating its protective role against Ang II-induced aging, potentially through the regulation of SIRT1 activity [29]. TSG also mitigated TNF-α-mediated cell damage in HUVECs by inhibiting vimentin expression via the TGFβ/Smad signaling pathway [51]. Furthermore, TSG rejuvenated aging hematopoietic stem cells (HSCs), particularly those predisposed to lymphoid differentiation, by modulating the AMPK-Tet2 axis. TSG treatment obviously increased the absolute number of common lymphoid progenitors (CLPs) and B lymphocytes, facilitating the repopulation potential of aging HSCs/CLPs in mice. This rejuvenation was linked to epigenetic reprogramming, which enhanced regenerative abilities and lymphopoiesis [28].

In conclusion, TSG demonstrates significant potential in alleviating vascular senescence and AS through multiple mechanisms, including the inhibitions of inflammation, oxidative stress, and lipid accumulation; the enhancements of telomerase activity, mitochondrial function, and epigenetic reprogramming; and the modulations of intestinal microbiota and immune responses. These findings position TSG as a promising candidate for developing therapies targeting AS and other age-related vascular diseases.

#### 3.3.2. Anti-Hypertensive Effects

Hypertension is a leading global risk factor for cardiovascular diseases, strokes, and mortality, affecting approximately 1.28 billion individuals aged 30 to 79 worldwide [52]. Age is a significant contributor to the development of hypertension, cardiovascular diseases, and cognitive impairment [53]. TSG demonstrated vasodilatory effects in U46619-induced contractions of the rat superior mesenteric artery (SMA) by activating the SIRT1 pathway and inhibiting thromboxane prostanoid receptors [30]. Furthermore, TSG partially ameliorated microvascular endothelial dysfunction through the activation of the Akt/mTOR pathway, which suppressed autophagy [54]. This suggests its utility in preventing subclinical vascular alterations associated with prehypertension. In hyperhomocysteinemia (HHcy) models, TSG effectively lowered blood pressure (BP) and decreased plasma Hcy and endothelin-1 (ET-1) levels by inhibiting the ERK_1/2_/NF-κB/ET_B2_ signaling pathway, as evidenced by both in vivo and in vitro studies [31].

In studies utilizing Zucker diabetic fatty (ZDF) rats and omentin-1 knockout (OMT^−/−^) mice, along with co-culture systems of HUVECs and mature adipocytes, TSG reduced systolic BP and improved endothelial vasodilation. Omentin-1 downregulation is associated with endothelial dysfunction and hypertension in obese individuals. TSG treatment increased omentin-1 levels by enhancing peroxisome proliferator-activated receptor-γ (PPAR-γ) binding to the *Itln-1* promoter in adipose tissue. This mechanism contributed to endothelial protection by activating the Akt/eNOS/NO pathway and alleviating oxidative and nitrative stress [32].

Taken together, TSG exhibits significant potential as an antihypertensive agent by improving endothelial function, reducing oxidative stress, and modulating key signaling pathways involved in vascular regulation. These properties make TSG a promising candidate for addressing hypertension and related cardiovascular diseases, particularly in the context of aging and obesity.

### 3.4. Reproductive Protective Effects of TSG

TSG exhibits potential in delaying testicular senescence and protecting male gonadal function. In a rat model of testicular Leydig cell aging induced by H_2_O_2_ + FeSO_4_, TSG significantly downregulated the expression of SA-β-gal and insulin-like growth factor binding protein 3 (IGFBP3), while upregulating levels of IGF-1, insulin receptor (INSR), insulin receptor substrate 1 (IRS1), and IRS2 [33]. These findings suggest that TSG delays Leydig cell aging by modulating the insulin/IGF-1 signaling pathway and exerting antioxidant effects.

Additionally, TSG demonstrates protective effects against ovarian aging. In both young and aged mice, TSG treatment distinctly preserved oocyte quantity and quality, attenuated the decline of cytochrome P450 enzymes (CYP11a and CYP19), which are critical for sex hormone synthesis, and maintained high levels of estrogen receptor beta (ER-β), thereby enhancing estrogen sensitivity. TSG also upregulated mitochondrial biogenesis-related genes (*pgc1α* and *atp6*) and increased anti-Müllerian hormone (AMH) levels in aged mice, a key biomarker of ovarian function [34]. Furthermore, studies using an E-screen assay revealed that TSG significantly promoted the proliferation of ER (+) human breast cancer MCF-7 cells and upregulated the expression of estrogen-dependent genes, including *ERα*, *ERβ*, and *pS2*, indicating its estrogenic activity [35].

Collectively, these findings suggest that TSG may serve as a potential phytoestrogen for treating conditions such as premature ovarian failure (POF), premature ovarian insufficiency (POI), diminished ovarian reserve (DOR), and estrogen deficiency-related disorders, including menopausal syndrome and postmenopausal osteoporosis (PMOP). However, further research is needed to confirm these therapeutic potentials.

### 3.5. Bone Protective Effects of TSG

Osteoporosis (OP) is a systemic skeletal disease characterized by reduced bone mineral density (BMD), increased bone fragility, and a heightened risk of fractures [55]. Aging and estrogen deficiency are primary contributors to OP pathogenesis. In a study utilizing ovariectomized (OVX) rats as an OP model and H_2_O_2_ to induce oxidative stress and dysfunction in MC3T3-E1 cells, TSG was found to significantly mitigate bone loss. It increased the levels of bone formation markers, including connectivity density (Conn.D), trabecular number (Tb.N), BMD, and glutathione peroxidase (GSH-Px) in bone tissue. Additionally, TSG enhanced the expression of alkaline phosphatase (ALP), osteopontin (OPN), osteocalcin (OCN), collagen I, and calcium deposition in MC3T3-E1 cells [36]. These effects are potentially mediated through the inhibition of miR-34a and the upregulation of SIRT1.

Moreover, TSG demonstrated beneficial effects on organ weight and bone length in estrogen-deficient conditions. In OVX mice, TSG treatment increased serum bone alkaline phosphatase (BALP) levels and reduced tartrate-resistant acid phosphatase (TRAP) activity, thereby attenuating bone loss and inhibiting bone destruction [56]. In a streptozotocin-induced diabetic OP mouse model, TSG significantly elevated calcium content in both serum and bone, improved trabecular bone microarchitecture, and increased the osteoprotegerin (OPG) to receptor activator of nuclear factor kappa B ligand (RANKL) ratio, indicating its protective role in diabetic OP [37].

In MC3T3-E1 cells, TSG treatment upregulated the expression of OPG, cyanate, runt-related transcription factor 2 (RUNX-2), osterix, and collagen type I α1, while downregulating RANKL and macrophage colony-stimulating factor (M-CSF). This activation of the PI3K/Akt pathway promoted osteoblast proliferation and differentiation [57]. Furthermore, TSG enhanced ALP activity and OCN content in rat mesenchymal stem cells (MSCs) and protected against bone loss in dexamethasone-induced zebrafish [58]. It also obviously promoted the proliferation of bone marrow MSCs and upregulated osteogenic differentiation markers, including ALP, OCN, Col1a1, RUNX-2, and β-catenin [38].

In summary, TSG exhibits potent anti-osteoporotic effects by reducing bone resorption and enhancing bone formation. These mechanisms may be linked to its estrogenic activity, warranting further investigation.

### 3.6. Other Protective Effects of TSG

TSG has demonstrated potential in improving the physiological functions of aged mice subjected to excessive caloric intake and in delaying the onset of senescence-related symptoms. The anti-aging effects of TSG were mediated, at least partially, through the AMPK/SIRT1/PGC-1α pathway. This mechanism leads to significant improvements in motor function, bone mineral density, and the mitigation of high-calorie-induced organ pathology, such as liver and kidney damage, as well as the enhancement of mitochondrial function [59]. These findings suggest that TSG could serve as a promising therapeutic candidate for addressing aging-related disorders and complications arising from excessive caloric intake.

In a cisplatin-induced myelosuppressive rat model, TSG was found to promote the proliferation of BMSCs and increase peripheral white blood cell counts following chemotherapy. This effect might be attributed to the inhibition of cyclin-dependent kinase inhibitor 1A (CDKN1A) overexpression and the upregulation of cyclinE1 [60]. In vitro study further supported TSG’s protective role against cisplatin-induced injuries, preserving both osteogenic and adipogenic differentiation in BMSCs [60].

Additionally, TSG exhibits significant effects on hair regrowth. Depilated mice treated with TSG demonstrated marked hair regrowth, which was associated with the inhibition of apoptotic factors, including Fas, p53, Bax, active caspase-3, and procaspase-9 activities [61]. Furthermore, a systematic review showed that TSG might act as a key molecular component of *P. multiflorum* in protecting against age-related hearing loss [62].

Despite the growing body of evidence highlighting TSG’s anti-aging properties and potential therapeutic applications, the intricate pharmacological mechanisms underlying these effects remain incompletely understood. Further research is needed to elucidate these mechanisms and expand the clinical significance of TSG in addressing aging-related conditions.

### 3.7. Effects of Other P. multiflorum Extracts Against Aging and Age-Related Diseases

#### 3.7.1. Identified Compounds

Emodin, an anthraquinone in *P. multiflorum*, also demonstrates neuroprotection by modulating apoptosis-related proteins (Bcl-2, Bax, caspase-3) and inhibiting the HDAC4/JNK pathway, thereby alleviating diabetic cognitive impairment [14,63]. It also exhibits anti-atherosclerotic effects via PI3K/AKT/mTOR suppression [64] and promotes hair darkening through MITF-mediated upregulation of tyrosinase [65]. Physcion, another anthraquinone, inhibits 5α-reductase, improving hair follicle regeneration in androgenic alopecia [66]. Mito-TSGs (TSG derivatives) enhance mitochondrial function, showing promise in AD models by reducing oxidative stress [67]. Polydatin attenuates bone loss by suppressing MAPK signaling [68], while polysaccharides extend the lifespan in *C. elegans* via oxidative stress mitigation [69].

#### 3.7.2. Ethanol Extract

The 75% ethanol extract from *P. multiflorum* regulates DNA methylation [70] and improves lipid metabolism [71] in D-gal-stimulated aging mice. And it also alleviates glucocorticoid-induced OP by enhancing autophagy in aging mice [72]. A 50% ethanol extract ameliorates vascular dementia by restoring vitamin B6 and taurine metabolic pathways [73], while a 60% fraction extends *C. elegans* lifespan via DAF-16/SIR-2.1/SKN-1 activation [74]. Additionally, an 80% methanol extract exhibits phytoestrogenic activity, boosting MCF7 proliferation [35].

#### 3.7.3. Aqueous Extract

The aqueous extract from *P. multiflorum* significantly mitigates diabetic encephalopathy by inhibiting HDAC4/JNK-induced apoptosis [63] and enhances cognition in aging mice via oxidative stress reduction [75]. It also displays estrogen-like effects, promoting uterine growth [76], and improves androgenic alopecia with mechanisms linked to reduced androgen levels and enhanced Wnt/β-catenin signaling [77]. An unidentified *P. multiflorum* component delays skin aging by enhancing collagen synthesis and mitophagy [78].

Supplementary Table S2 summarizes the recent studies (2020–2024) investigating the effects of other *P. multiflorum* extracts against aging and age-related diseases. While these *P. multiflorum* extracts also show broad anti-aging potential, the specific bioactive components warrant further isolation and mechanistic validation.

## 4. Conclusions and Future Perspectives

The aging population represents a significant global challenge, underscoring the urgent need for the development of anti-aging therapeutics aimed at extending healthy lifespans. This issue is of paramount importance to both the medical community and society at large. TSG, the primary quality marker of *P. multiflorum*, demonstrates unique potential in preventing and treating aging and age-related diseases. Our findings reveal that TSG exhibits a broad spectrum of anti-aging effects, including lifespan extension, neuroprotection (e.g., ameliorating AD and PD), cardiovascular protection (e.g., alleviating vascular senescence, AS, and hypertension), delay of gonadal aging, reduction in bone loss (e.g., mitigating OP), and promotion of hair regrowth, among others. Mechanistically, TSG exerts its anti-aging effects by mitigating oxidative stress and inflammation, suppressing cellular apoptosis, promoting mitophagy, enhancing mitochondrial function and telomerase activity, and regulating methylation and intestinal flora. This study further demonstrates that TSG therapeutically targets multiple hallmarks of aging, including cellular senescence, chronic inflammation, impaired macroautophagy, mitochondrial dysfunction, telomere attrition, epigenetic alterations, deregulated nutrient sensing, and dysbiosis, thereby slowing, halting, or even reversing senescence. Other *P. multiflorum* extracts exhibit similar effects and mechanisms. These effects align with the holistic principles of traditional Chinese medicine.

However, several limitations must be addressed to advance this field. (1) Limited clinical evidence: Most current studies are preclinical, relying on animal or cell models. This lack of robust clinical evidence hinders the translation of TSG’s potential into practical applications. Future research should prioritize high-quality, multicenter, large-sample clinical trials to validate the efficacy and safety of TSG, thereby facilitating its clinical adoption and guiding interventions for aging and age-related diseases. (2) Scope of research: While TSG has shown efficacy in improving conditions such as AD, PD, AS, and OP, its protective effects against other age-related diseases, such as DOR and PMOP, remain underexplored and require further investigation. (3) Compositional complexity: Beyond TSG and a few well-identified compounds, the complex composition of *P. multiflorum* complicates pharmacological studies. Incomplete component analyses obscure active constituents and their mechanisms. Further research should identify key components and their interactions with aging hallmarks to reveal therapeutic targets. (4) Regional bias: Although this review included randomized controlled trials without regional restrictions, the majority of the studies analyzed were conducted in China. This may introduce regional biases, potentially due to the limited global promotion and application of TSG. A critical challenge is how to effectively integrate TSG into the clinical management of aging and age-related diseases on a global scale. Addressing these limitations through the proposed research directions (points 1–3) is expected to establish a quantitative system for evaluating TSG’s efficacy. Such efforts will systematically elucidate the scientific basis of TSG’s anti-aging effects, promote its widespread application, and contribute to the goal of achieving healthy longevity.

## Supplementary Materials

The following supporting information can be downloaded at https://www.mdpi.com/article/10.3390/ijms26073381/s1.

## Abbreviations

The following abbreviations are used in this manuscript:

Aβ	β-Amyloid
AD	Alzheimer’s disease
AKT	Protein kinase B
Ang II	Angiotensin II
APP	Amyloid precursor protein
AS	Atherosclerosis
BALP	Bone alkaline phosphatase
Bcl-2	B-cell lymphoma-2
Bax	Bcl-2-associated X protein
BDNF	Brain-derived neurotrophic factor
BMD	Bone mineral density
BMSCs	Bone mesenchymal stem cells
BP	Blood pressure
CDKN1A	Cyclin-dependent kinase inhibitor 1A
*C. elegans*	*Caenorhabditis elegans*
cGAS	Cyclic GMP-AMP synthase
CLPs	Common lymphoid progenitors
Conn.D	Connectivity density
DA	Dopaminergic
DAF-16	Abnormal dauer formation-16
DCs	Dendritic cells
DE	Diabetic encephalopathy
D-gal	D-galactose
DOR	Diminished ovarian reserve
ERK	Extracellular signal-regulated kinase
ET-1	Endothelin-1
FGF2	Fibroblast growth factor 2
FOXO	Forkhead box O
GSH-Px	Glutathione peroxidase
GSK-3β	Glycogen synthase kinase-3β
HAECs	Human aortic endothelial cells
HDAC	Histone deacetylase
HHcy	Hyperhomocysteinemia
HSCs	Hematopoietic stem cells
HUVECs	Human umbilical vein endothelial cells
IGF	Insulin-like growth factor
IRS1	Insulin receptor substrate 1
JNK	Jun N-terminal kinase
LPS	Lipopolysaccharide
MAPK	Mitogen-activated protein kinase
MCP-1	Monocyte chemotactic protein-1
MMP	Mitochondrial membrane potential
MPTP	1-Methyl-4-phenyl-1, 2, 3, 6-tetrahydropyridine
MPP+	1-Methyl-4-phenylpyridinium
mTOR	Mechanistic target of rapamycin
NLRP3	Nod-like receptor protein 3
Nrf2	Nuclear factor erythroid 2-related factor 2
NSCs	Neural stem cells
OCN	Osteocalcin
OPG	Osteoprotegerin
OPN	Osteopontin
OVX	Ovariectomized
PACS-2	Phosphofurin acidic cluster sorting protein-2
PAI-1	Plasminogen activator inhibitor-1
PD	Parkinson’s disease
PGC-1α	Proliferator-activated receptor γ coactivator 1α
PI3K	Phosphatidylinositol-3-kinase
PMOP	Postmenopausal osteoporosis
POF	Premature ovarian failure
POI	Premature ovarian insufficiency
*P.**multiflorum*	*Polygonum multiflorum* Thunb.
PPAR-γ	Peroxisome proliferator-activated receptor-γ
RANKL	Receptor activator for nuclear factor kappa B ligand
ROS	Reactive oxygen species
RUNX-2	Runt-related transcription factor 2
SA-β-gal	Senescence-associated β-galactosidase
SIRT1	Silent information regulator 1
SKN-1	Skinhead-1
SOD	Superoxide dismutase
STING	Stimulator of interferon genes
Tb.N	Trabecular number
TC	Total cholesterol
TG	Triglyceride
TNF-α	Tumor necrosis factor-α
TRAP	Tartrate-resistant acid phosphatase
TrkB	Tropomyosin receptor kinase-B
TSG	2,3,5,4′-Tetrahydroxystilbene-2-O-β-D-glucoside
VaD	Vascular dementia
VCAM-1	Vascular cell adhesion molecule-1
ZDF	Zucker diabetic fatty
6-OHDA	6-Hydroxydopamine

## References

[1] Stakos D.A., Stamatelopoulos K., Bampatsias D., Sachse M., Zormpas E., Vlachogiannis N.I., Tual-Chalot S., Stellos K. (2020). The Alzheimer’s Disease Amyloid-Beta Hypothesis in Cardiovascular Aging and Disease: JACC Focus Seminar. J. Am. Coll. Cardiol..

[2] López-Otín C., Pietrocola F., Roiz-Valle D., Galluzzi L., Kroemer G. (2023). Meta-hallmarks of aging and cancer. Cell Metab..

[3] López-Otín C., Blasco M.A., Partridge L., Serrano M., Kroemer G. (2013). The hallmarks of aging. Cell.

[4] López-Otín C., Blasco M.A., Partridge L., Serrano M., Kroemer G. (2023). Hallmarks of aging: An expanding universe. Cell.

[5] Zhao Y., Simon M., Seluanov A., Gorbunova V. (2023). DNA damage and repair in age-related inflammation. Nat. Rev. Immunol..

[6] Wu Z., Qu J., Zhang W., Liu G.H. (2024). Stress, epigenetics, and aging: Unraveling the intricate crosstalk. Mol. Cell.

[7] Li X., Li C., Zhang W., Wang Y., Qian P., Huang H. (2023). Inflammation and aging: Signaling pathways and intervention therapies. Signal Transduct. Target. Ther..

[8] Commission C.P. (2020). Pharmacopoeia of the People’s Republic of China 2020.

[9] Xia H., Cheng X., Cao M., Sun X., He F., Yao X., Liu H. (2023). Tetrahydroxystilbene Glucoside Attenuates Oxidative Stress-Induced Aging by Regulating Oxidation Resistance and Inflammation in Larval Zebrafish. Zebrafish.

[10] Sun M., Wei C., Gao Y., Chen X., Zhong K., Li Y., Yang Z., Gao Y., Wang H. (2024). TSG Extends the Longevity of Caenorhabditis elegans by Targeting the DAF-16/SKN-1/SIR-2.1-Mediated Mitochondrial Quality Control Process. Antioxidants.

[11] Miao B.B., Gao D., Hao J.P., Li Y.L., Li L., Wang J.B., Xiao X.H., Yang C.C., Zhang L. (2022). Tetrahydroxy stilbene glucoside alters neurogenesis and neuroinflammation to ameliorate radiation-associated cognitive disability via AMPK/Tet2. Int. Immunopharmacol..

[12] Gao Y., Li J., Li J., Hu C., Zhang L., Yan J., Li L., Zhang L. (2020). Tetrahydroxy stilbene glycoside alleviated inflammatory damage by mitophagy via AMPK related PINK1/Parkin signaling pathway. Biochem. Pharmacol..

[13] Kang B.Q., Li Y., He X.X., Xiao Z., Hu R., Luo C.L., Qiao M.Y., Wu G.Y., Li Z.Z., Zhu X.Y. (2024). Mechanism of stilbene glycosides on apoptosis of SH⁃SY5Y cells via regulating PI3K/AKT signaling pathway. J. Hainan Med. Univ..

[14] Chen G., Xu Y.J., Huang C.X.D., Lin H.R., Yang T.T., Zhu L.Y., Li X., Pan W. (2022). Tetrahydroxystilbene glucoside and emodin improves the hippocampal neuronal apoptosis in mice induced by high glucose. Tianjin Med. J..

[15] Gao D., Hao J.P., Li B.Y., Zheng C.C., Miao B.B., Zhang L., Li Y.L., Li L., Li X.J., Zhang L. (2023). Tetrahydroxy stilbene glycoside ameliorates neuroinflammation for Alzheimer’s disease via cGAS-STING. Eur. J. Pharmacol..

[16] Wu W.X., Su Y.Z., Li Z.Z., Liu C.Y., Meng W.Y., Huang J., Zhu X.Y., Liu J.B., Liu D.M., Huang Z.S. (2022). Effects of stilbene glycoside on CDK5, MAPK1 and PP1 in Alzheimer’s disease mice. Chin. J. Gerontol..

[17] Xia X.Y., Li Y.B., He X.X., Li Y., Kang B.Q., Li Z.Z., Huang Z.S. (2023). Effect of stilbene glycoside on Ser199 posphorylation in Alzheimer’s disease rats. Chin. J. Clin. Pharmacol..

[18] Li Y.B. (2023). Study on the Intervention of Stilbene Glycoside on Tau Protein Phosphorylation in Rats with Alzheimer’s Disease via PI3K/AKT/GSK-3β Signaling Pathway. Master’s Thesis.

[19] Li Y., Kang B.Q., He X.X., Hu R., Xiao Z., Luo C.L., Wu G.Y., Huang Z.S. (2023). The effect and mechanism of stilbene glycosides on improving neuronal injury in Alzheimer’s disease rats by regulating ASK/MKK7/JNK pathway. J. Hainan Med. Univ..

[20] Wang Y.Y., Ye Q.Y., Qian J., Liu Z.P., Luo H.B., Li Y. (2024). Effect of PACS-2 on the development of Alzheimer’s disease. China Mod. Dr..

[21] Gao Y., Li J., Hu K., Wang S., Yang S., Ai Q., Yan J. (2024). Phosphoproteomic analysis of APP/PS1 mice of Alzheimer’s disease by DIA based mass spectrometry analysis with PRM verification. J. Proteom..

[22] Yu Y., Lang X.Y., Li X.X., Gu R.Z., Liu Q.S., Lan R., Qin X.Y. (2019). 2,3,5,4’-Tetrahydroxystilbene-2-O-β-d-glucoside attenuates MPP+/MPTP-induced neurotoxicity in vitro and in vivo by restoring the BDNF-TrkB and FGF2-Akt signaling axis and inhibition of apoptosis. Food Funct..

[23] Zhang L., Yang H. (2021). Promotive effects of tetrahydroxystilbene glucoside on the differentiation of neural stem cells from the mesencephalon into dopaminergic neurons. Neurosci. Lett..

[24] Li F., Zhang T., He Y., Gu W., Yang X., Zhao R., Yu J. (2020). Inflammation inhibition and gut microbiota regulation by TSG to combat atherosclerosis in ApoE(-/-) mice. J. Ethnopharmacol..

[25] Yang Y., Bai D., Jiang L., Chen Y., Wang M., Wang W., Wang H., He Q., Bu G., Long J. (2024). Stilbene glycosides alleviate atherosclerosis partly by promoting lipophagy of dendritic cells. Int. Immunopharmacol..

[26] Li M., Meng Y., Hong X., Chai H., Huang J., Wang F., Zhang W., Wang J., Liu Q., Xu Y. (2024). Anti-atherosclerotic effect of tetrahydroxy stilbene glucoside via dual-targeting of hepatic lipid metabolisms and aortic M2 macrophage polarization in ApoE(-/-) mice. J. Pharm. Biomed. Anal..

[27] Wang C.Y., Wang J., Cao J., Xu J., Wu R.M., Xu X.L. (2022). Activating PGC-1α-mediated signaling cascades in the aorta contributes to the amelioration of vascular senescence and atherosclerosis by 2,3,4’,5-tetrahydroxystilbene-2-O-β-d-glycoside. Phytomedicine.

[28] Gao D., Yi W.W., Liu B., Zhang C.E., Yang C.C., Zeng L., Li L., Luo G., Zhang L., Ju Z.Y. (2024). Tetrahydroxy stilbene glucoside rejuvenates aging hematopoietic stem cells with predilection for lymphoid differentiation via AMPK and Tet2. J. Adv. Res..

[29] Fan W., Guo Y., Cao S., Cao S., Xie Y., Liu X., Jin B. (2021). Tetrahydroxystilbene glucoside alleviates angiotensin II induced HUVEC senescence via SIRT1. Can. J. Physiol. Pharmacol..

[30] Chen Y.L., Qin Q.H., Hou Y., Zhang H., Jia M. (2022). Study on the mechanism of stilbene glycosides vasodilation via SIRT1-TP pathway based on molecular docking technology. Chin. J. Hosp. Pharm..

[31] Jia M., Su X., Qin Q., Li Y., Wang S., Chen Y. (2022). Tetrahydroxystilbene glucoside attenuated homocysteine-upregulated endothelin receptors in vascular smooth muscle cells via the ERK(1) (/2) /NF-κB signaling pathway. Phytother. Res..

[32] Dong Q., Xing W., Li K., Zhou X., Wang S., Zhang H. (2021). Tetrahydroxystilbene glycoside improves endothelial dysfunction and hypertension in obese rats: The role of omentin-1. Biochem. Pharmacol..

[33] Li S.H., Jiang L.P., Niu J.Y., Wang L., Niu S.Y., Qi F. (2021). Regulation mechanism of tetrahydroxy stilbene glycoside on insulin / IGF-1 signaling pathway to delaying the senescence of rat leydig cells. J. Hebei Univ. (Nat. Sci. Ed.).

[34] Lin H.Y., Yang Y.N., Chen Y.F., Huang T.Y., Crawford D.R., Chuang H.Y., Chin Y.T., Chu H.R., Li Z.L., Shih Y.J. (2022). 2,3,5,4’-Tetrahydroxystilbene-2-O-β-D-Glucoside improves female ovarian aging. Front. Cell Dev. Biol..

[35] Akter R., Yang D.U., Ahn J.C., Awais M., Nahar J., Ramadhania Z.M., Kim J.Y., Lee G.J., Kwak G.Y., Lee D.W. (2023). Comparison of In Vitro Estrogenic Activity of Polygoni multiflori Radix and Cynanchi wilfordii Radix via the Enhancement of ERα/β Expression in MCF7 Cells. Molecules.

[36] Wang Q., Yang P., Sun J.J., Yao S.H. (2022). Effect and mechanism of stilbene glucoside regulating miR-34a/SIRT1 on osteoporosis in rats. Chin. J. Osteoporos..

[37] Zhang J., Chen X., Chen B., Tong L., Zhang Y. (2019). Tetrahydroxy stilbene glucoside protected against diabetes-induced osteoporosis in mice with streptozotocin-induced hyperglycemia. Phytother. Res..

[38] Liang Y.Y., Zhen L., Chen X.L., Chen J.M., Cui L., Liu Y.Y. (2022). Effects and mechanism of tetrahydroxystilbene glycoside on the proliferation and differentiation of bone marrow mesenchymal stem cells. Chin. J. Hosp. Pharm..

[39] Büchter C., Zhao L., Havermann S., Honnen S., Fritz G., Proksch P., Wätjen W. (2015). TSG (2,3,5,4’-Tetrahydroxystilbene-2-O-β-D-glucoside) from the Chinese Herb Polygonum multiflorum Increases Life Span and Stress Resistance of Caenorhabditis elegans. Oxid. Med. Cell Longev..

[40] Zhou X.X. (2013). Study on the Preparation Process of High Purity Tetrahydroxystilbene Glucoside and Its Anti-Aging Mechanism. Ph.D. Thesis.

[41] Wu D.J., Fan F., Chen J.Y., Ge Z.L. (2022). Effect of Gstm3 on Tetrahydroxy stilbene glycoside delaying senescence of SH-SY5Y cells. J. Zunyi Med. Univ..

[42] Lin F., Zhou Y., Shi W., Wan Y., Zhang Z., Zhang F. (2016). Tetrahydroxystilbene Glucoside Improves Neurotrophic Factors Release in Cultured Astroglia. CNS Neurol. Disord. Drug Targets.

[43] Monteiro A.R., Barbosa D.J., Remião F., Silva R. (2023). Alzheimer’s disease: Insights and new prospects in disease pathophysiology, biomarkers and disease-modifying drugs. Biochem. Pharmacol..

[44] Zhang R.Y., Zhang L., Zhang L., Wang Y.L., Li L. (2018). Anti-amyloidgenic and neurotrophic effects of tetrahydroxystilbene glucoside on a chronic mitochondrial dysfunction rat model induced by sodium azide. J. Nat. Med..

[45] Zeng C.H., Cao Z.Y., Chi X.Y., Duan Y.T., Zhou F.L., Liu S.L., Yang K. (2023). Action mechanism of radix polygoni multiflori in the improvement of Alzheimer’s disease based on network pharmacology and molecular docking. Chin. J. Gerontol..

[46] Leite Silva A.B.R., Gonçalves de Oliveira R.W., Diógenes G.P., de Castro Aguiar M.F., Sallem C.C., Lima M.P.P., de Albuquerque Filho L.B., Peixoto de Medeiros S.D., Penido de Mendonça L.L., de Santiago Filho P.C. (2023). Premotor, nonmotor and motor symptoms of Parkinson’s Disease: A new clinical state of the art. Ageing Res. Rev..

[47] Zhang R., Sun F., Zhang L., Sun X., Li L. (2017). Tetrahydroxystilbene glucoside inhibits α-synuclein aggregation and apoptosis in A53T α-synuclein-transfected cells exposed to MPP. Can. J. Physiol. Pharmacol..

[48] Huang C., Lin F., Wang G., Lu D., Wu Q., Liu J., Shi J., Zhang F. (2018). Tetrahydroxystilbene Glucoside Produces Neuroprotection against 6-OHDA-Induced Dopamine Neurotoxicity. Oxid. Med. Cell Longev..

[49] Ungvari Z., Tarantini S., Sorond F., Merkely B., Csiszar A. (2020). Mechanisms of Vascular Aging, A Geroscience Perspective: JACC Focus Seminar. J. Am. Coll. Cardiol..

[50] Yi B., Nguyen M.C., Won M.H., Kim Y.M., Ryoo S. (2017). Arginase Inhibitor 2,3,5,4’-Tetrahydroxystilbene-2-O-β-D-Glucoside Activates Endothelial Nitric Oxide Synthase and Improves Vascular Function. Planta Med..

[51] Yao W., Gu C., Shao H., Meng G., Wang H., Jing X., Zhang W. (2015). Tetrahydroxystilbene glucoside improves TNF-α-induced endothelial dysfunction: Involvement of TGFβ/Smad pathway and inhibition of vimentin expression. Am. J. Chin. Med..

[52] (NCD-RisC) N.R.F.C. (2021). Worldwide trends in hypertension prevalence and progress in treatment and control from 1990 to 2019: A pooled analysis of 1201 population-representative studies with 104 million participants. Lancet.

[53] Hay M., Barnes C., Huentelman M., Brinton R., Ryan L. (2020). Hypertension and Age-Related Cognitive Impairment: Common Risk Factors and a Role for Precision Aging. Curr. Hypertens. Rep..

[54] Dong Q., Xing W., Fu F., Liu Z., Wang J., Liang X., Zhou X., Yang Q., Zhang W., Gao F. (2016). Tetrahydroxystilbene Glucoside Inhibits Excessive Autophagy and Improves Microvascular Endothelial Dysfunction in Prehypertensive Spontaneously Hypertensive Rats. Am. J. Chin. Med..

[55] Reid I.R., Billington E.O. (2022). Drug therapy for osteoporosis in older adults. Lancet.

[56] Kim S.J., Hwang Y.H., Mun S.K., Hong S.G., Kim K.J., Kang K.Y., Son Y.J., Yee S.T. (2018). Protective Effects of 2,3,5,4’-Tetrahydroxystilbene-2-O-β-d-glucoside on Ovariectomy Induced Osteoporosis Mouse Model. Int. J. Mol. Sci..

[57] Fan Y.S., Li Q., Hamdan N., Bian Y.F., Zhuang S., Fan K., Liu Z.J. (2018). Tetrahydroxystilbene Glucoside Regulates Proliferation, Differentiation, and OPG/RANKL/M-CSF Expression in MC3T3-E1 Cells via the PI3K/Akt Pathway. Molecules.

[58] Zheng Y., Li J., Wu J., Yu Y., Yao W., Zhou M., Tian J., Zhang J., Cui L., Zeng X. (2017). Tetrahydroxystilbene glucoside isolated from Polygonum multiflorum Thunb. demonstrates osteoblast differentiation promoting activity. Exp. Ther. Med..

[59] Ning Z., Li Y., Liu D., Owoicho Orgah J., Zhu J., Wang Y., Zhu Y. (2018). Tetrahydroxystilbene Glucoside Delayed Senile Symptoms in Old Mice via Regulation of the AMPK/SIRT1/PGC-1α Signaling Cascade. Gerontology.

[60] Xu X.G. (2018). Study on the Mechanism of Kidney-Tonifying Method Antagonizing Myelosuppression After Chemotherapy by Regulating Bone Marrow Stem Cells. Master’s Thesis.

[61] Chen L., Duan H., Xie F., Gao Z., Wu X., Chen F., Wu W. (2018). Tetrahydroxystilbene Glucoside Effectively Prevents Apoptosis Induced Hair Loss. Biomed. Res. Int..

[62] Hu S., Sun Q., Xu F., Jiang N., Gao J. (2023). Age-related hearing loss and its potential drug candidates: A systematic review. Chin. Med..

[63] Xu Y., Li H., Chen G., Zhu L., Lin H., Huang C., Wei S., Yang T., Qian W., Li X. (2022). Radix polygoni multiflori protects against hippocampal neuronal apoptosis in diabetic encephalopathy by inhibiting the HDAC4/JNK pathway. Biomed. Pharmacother..

[64] Zhang L., Tian W.Y., Wang H.S., Lai C.Q., Li M.J., Xia L., Leng C.L. (2021). Shouwu emodin against ApoE-/- mouse atherosclerosis based on PI3K/AKT/mTOR signaling pathway. Chin. J. Gerontol..

[65] Kim J., Kim M.M. (2022). The effect of emodin on melanogenesis through the modulation of ERK and MITF signaling pathway. Nat. Prod. Res..

[66] Lao Z., Fan Y., Huo Y., Liao F., Zhang R., Zhang B., Kong Z., Long H., Xie J., Sang C. (2022). Physcion, a novel inhibitor of 5α-reductase that promotes hair growth in vitro and in vivo. Arch. Dermatol. Res..

[67] Qian J., Li Y., Wang Y., Ye Q., Luo H. (2023). Effects of tetrahydroxy stilbene glycoside derivatives on free radical damage and apoptosis in APP695V717I transgenic mice. Redox Rep..

[68] Lin Z., Xiong Y., Hu Y., Chen L., Panayi A.C., Xue H., Zhou W., Yan C., Hu L., Xie X. (2021). Polydatin Ameliorates Osteoporosis via Suppression of the Mitogen-Activated Protein Kinase Signaling Pathway. Front. Cell Dev. Biol..

[69] Fan J., Wang Y., Yang J., Gu D., Kang S., Liu Y., Jin H., Wei F., Ma S. (2024). Anti-aging activities of neutral and acidic polysaccharides from Polygonum multiflorum Thunb in Caenorhabditis elegans. Int. J. Biol. Macromol..

[70] Zou J.Y., Yang J.Q., Xu L., Fan F., Ge Z.L. (2020). Effects of Polygonum multiflorum extract on DNA methylation and gene transcription in brain tissue of aging mice. J. Third Mil. Med. Univ..

[71] Yang J., He Y., Zou J., Xu L., Fan F., Ge Z. (2019). Effect of Polygonum Multiflorum Thunb on liver fatty acid content in aging mice induced by D-galactose. Lipids Health Dis..

[72] Liu Y., Zhou M., Wang R., Liang Y., Zhuang G., Chen X., Luo S., Cai Y., Song C., Liu L. (2024). Alleviation of Glucocorticoid-Induced Osteoporosis in Rats by Ethanolic Reynoutria multiflora (Thunb.) Moldenke Extract. J. Med. Food.

[73] Wu F., Li Y., Liu W., Xiao R., Yao B., Gao M., Xu D., Wang J. (2022). Comparative Investigation of Raw and Processed Radix Polygoni Multiflori on the Treatment of Vascular Dementia by Liquid Chromatograph-Mass Spectrometry Based Metabolomic Approach. Metabolites.

[74] Sun M.L., Chen X.Y., Cao J.J., Cui X.H., Wang H.B. (2021). Polygonum multiflorum Thunb extract extended the lifespan and healthspan of Caenorhabditis elegans via DAF-16/SIR-2.1/SKN-1. Food Funct..

[75] Ren J., Jiang Y.H., Liu S.J., Yu F., Pang H.M. (2024). Anti-aging effects of different drying methods of Polygonum multiflorum on D-galactose-induced aging mice. Pharm. Clin. Res..

[76] Zhu C., Li Y.F., Peng F., Yang C.F., Fu Y.Y., Tian M. (2020). Study on Phytoestrogen-like Effect and Mechanism of Polygoni Multiflori Radix Preparata. Chin. Arch. Tradit. Chin. Med..

[77] Pan F.Z., Chen M.X., Yi B., Xue Y.H., Wang Q.P., Wu F.Y., Ji E.H., Wu H.W., Xu J. (2024). Stewed Polygoni Multiflori Radix Treats androgenic Alopecia in Mice by Activating Wnt/β-catenin Signaling Pathway. Chin. J. Exp. Tradit. Med. Formulae.

[78] Liu X., Yang C., Deng Y., Liu P., Yang H., Du X., Du Y. (2021). Polygoni Multiflori Radix Preparat Delays Skin Aging by Inducing Mitophagy. Biomed. Res. Int..

