# Supplementary material for: 2,3,5,4′-Tetrahydroxystilbene-2-O-β-D-glucoside (TSG) from Polygonum multiflorum Thunb.: A Systematic Review on Anti-Aging
Source: Int J Mol Sci. 2025 Apr 4;26(7):3381. doi: 10.3390/ijms26073381 (PMC11989756; doi:10.3390/ijms26073381)
Supplement: Supplementary file 1 [file ijms-26-03381-s001.zip › Supplementary Table S1. Search strategy.pdf]

Table S1. Search strategy of PubMed and parallel to other databases

Database	Search strategy
	#1
PubMed	((((((((Polygonum multiflorum Thunb.[Title/Abstract]) OR (Polygonum multiflorum[Title/Abstract])) OR (Polygoni Multiflori Radix Praeparata[Title/Abstract])) OR (Polygoni Multiflori Radix[Title/Abstract])) OR (Heshouwu[Title/Abstract])) OR (Fallopia multiflora[Title/Abstract])) OR (TSG[Title/Abstract])) OR (2,3,5,4'-Tetrahydroxystilbene-2-O-β-d-glucoside[Title/Abstract])) OR (2,3,5,4'-Tetrahydroxyl diphenylethylene-2-O-glucoside[Title/Abstract])
	#2
	((((((((((((((Aging[Title/Abstract]) OR (Senescence[Title/Abstract])) OR (Biological Aging[Title/Abstract])) OR (Aging, Biological[Title/Abstract])) OR (Skin Aging[Title/Abstract])) OR (Cellular Senescence[Title/Abstract])) OR (Cognitive Aging[Title/Abstract])) OR (Immunosenescence[Title/Abstract])) OR (Longevity[Title/Abstract])) OR (lifespan[Title/Abstract])) OR (Degeneration[Title/Abstract])) OR (Alzheimer's disease[Title/Abstract])) OR (Parkinson's Disease[Title/Abstract])) OR (hypertension[Title/Abstract])) OR (Atherosclerosis[Title/Abstract])) OR (Osteoporosis[Title/Abstract])
	#3
	#1 AND #2
