# Supplementary material for: 2,3,5,4′-Tetrahydroxystilbene-2-O-β-D-glucoside (TSG) from Polygonum multiflorum Thunb.: A Systematic Review on Anti-Aging
Source: Int J Mol Sci. 2025 Apr 4;26(7):3381. doi: 10.3390/ijms26073381 (PMC11989756; doi:10.3390/ijms26073381)
Supplement: Supplementary file 1 [file ijms-26-03381-s001.zip › Supplementary Table S2. Summary of recent studies (2020--2024) on the effects of other extracts against aging and a.pdf]

Table S2. Summary of recent studies (2020–2024) on the effects of other extracts against aging and age-related diseases

Effects	Extracts of *P. multiflorum*	Aging model (inducer; object)	Treatment (dose; duration)	Component analysis	Potential mechanisms	Author (year)	References
**Lifespan extension**	**Polysaccharide**	Paraquat; *C. elegans*	2, 2.5, and 3.0 mg/mL; 48 h	UPLC-MS/MS	Oxidative stress↓ (NO↓, ROS↓, MDA↓, SOD↑, CAT↑, GSH-PX↑, lipofuscin↓)	Fan et al. (2024)	[69]
	**60% ethanol extract**	Paraquat, or Aβ transgenic *C. elegans* strain CL4176	50 μg/mL; 36 h, or 96 h	HPLC	Oxidative stress↓, age-related phenotypes↓, improving mitochondrial function (Insulin/IGF-1 pathway; MMP↑, ATP↑, ROS↓; lipofuscin↓; SIR-2.1↑, SKN-1↑; Aβ↓)	Sun et al. (2021)	[74]
**Neuroprotection**	**Emodin**	High-glucose; HT-22 cells	100 μM; 48 h	Unclear (standard)	Apoptosis↓ (HAT↓, HDAC↓, Bax↓, Caspase-3↓, Bcl-2↑)	Chen et al. (2022)	[14]
	**75% ethanol extract**	D-gal; C57BL/6 mice	0.3, 0.6, and 1 g/mL/kg; 60 days	None	Regulating methylation and transcription (SOD↑, GSH-Px↑, MDA↓, *Synpo*↑, *Mapkapk5*↑, *Tcf7*↑, *Rab15*↑)	Zou et al. (2020)	[70]
**Alleviating AD**	**Mito-TSGs**	AD model: APP695V717I transgenic mice	0.4 mL/20 g/d; 4 weeks	Unclear	Oxidative stress↓, apoptosis↓, mitochondrial function↑, and improving behavioral performance (H_2O_2↓, NO↓, MDA↓, LD↓, LDH↑, SOD↑, CAT↑, GSH-Px↑. MMP↑, caspase-3↓, Bax↓, Bcl-2↑)	Qian et al. (2023)	[67]
**Alleviating DE**	**Aqueous extract, and Emodin**	① DE model: Streptozotocin; SD rats. ② High glucose; HT-22 cells	① 1 and 2 g/kg/d; 12 weeks. ② Emodin 100 μM; 48 h	UPLC/ TQD IVD	Apoptosis↓, cell proliferation↑, cognitive function↑ (HDAC4/JNK pathway; HAT↓, p-JNK↓, HDAC↓, HDAC4↓, JNK↓, Bax↓, Caspase-3↓, Bcl-2↑)	Xu et al. (2022)	[63]
**Relieving VaD**	**50% ethanol extract**	VaD model: 2-VO; SD rats	2 g/kg/d; 4 weeks	LC–MS	Oxidative stress↓, ameliorating metabolism disordered (MDA↓, GSH↑)	Wu et al. (2022)	[73]
**Inhibiting AS**	**Emodin**	High-fat diet + LPS; ApoE$^{-/-}$ mice	40, 20 and 10 mg/kg; 6 weeks	Unclear (standard)	Autophagy↓ (PI3K/ AKT/mTOR pathway; PI3K↓, AKT↓, mTOR↓, TC↓, TG↓, LDL-C↓, HDL-C↑)	Zhang et al. (2021)	[64]
**Estrogenic activity**	**80% methanol extract**	ER (+) MCF-7 cells	100 nM; 24 h	HPLC	Inflammation↓, cell proliferation↑, acting as phytoestrogens (ER-α↑, ER-β↑, pS2↑, ROS↓, NO↓)	Akter et al. (2023)	[51]

Effects	Extracts of *P. multiflorum*	Aging model (inducer; object)	Treatment (dose; duration)	Component analysis	Potential mechanisms	Author (year)	References
	**Aqueous extract**	Infancy KM mice	1 and 4 g/kg/d; 5 days	None	Uterus growth↑ (E_2↑, ER-α↑, ER-β↑)	Zhu et al. (2020)	[76]
**Retarding liver aging**	**Aqueous extract**	D-gal; ICR mice	2 g/kg/d, 12 weeks	None	Oxidative stress↓, learning and memory↑, body weight↑, organ index↑, improving liver and kidney function (SOD↑, GSH-Px↑, MDA↓)	Ren et al. (2024)	[75]
**Reducing OP**	**Polydatin**	OP model: Dex; MC3T3-E1 cells	20, 40, and 80 μM; unclear	Unclear	Bone turnover markers↑, mineralization damage↓ (MAPK pathway; p-JNK↓, p-P38↓, p-ERK↓)	Lin et al. (2021)	[68]
	**75% ethanol extract**	GIOP model: Prednisone; SD rats	400 and 200 mg/kg/d; 21 weeks	HPLC	Apoptosis↓, autophagy↑, bone formation↑ (OCN↑, OPG↑, RANKL↑, TRACP-5b↓, CTX-I↓, LC3↑, Agt5↑, Beclin 1↑)	Liu et al. (2024)	[72]
**Promoting hair darkening**	**Emodin**	B16F1 cells	0.125-2 μM; 8 h	Unclear	Melanogenesis↑ (ERK pathway, MITF pathway; tyrosinase↑, TYR↑, TRP-1↑, TRP-2↑, MITF↑, SIRT1↑, ERK↓, FOXO1↓)	Kim et al. (2022)	[65]
**Promoting hair growth**	**Physcion**	Testosterone + depilated with wax/resin mixtures ; SD rats, C57BL/6 mice	5, 2 mg/d; at least 8 days	Purity > 98%	Improving hair follicle morphology, and increasing hair follicle count (5α-reductase↓)	Lao et al. (2022)	[66]
	**Aqueous extract**	Androgenetic alopecia model: Testosterone propionate; C57BL/6J mice	0.78, 1.56 and 3.12 g/kg/d; 60 days	None	New hair growth↑, melanin follicles↑ (Wnt/β-catenin pathway; β-catenin↑, p-GSK-3β↑, GSK-3β↓, T↓, DHT↓)	Pan et al. (2024)	[77]
**Delaying skin aging**	**Aqueous extract**	D-gal; mice (unclear)	40 g/kg/d; 10 weeks	Unclear	Oxidative damage↓, apoptosis↓, mitochondrial autophagy↑, fibrous tissue↑ (PINK1↑, Parkin↑, LC3 I / II ↑, p62↓; MMP↑, ATP ↑, ROS↓; Cleaved- cas3↓, Bax↓, Cyt-c↓, AIF↓, Smac↓, Bcl-2↑; type I/type III collagen↑)	Liu et al. (2021)	[78]
